# Uses of psychotropic drugs by university students in Switzerland

**DOI:** 10.1371/journal.pone.0305392

**Published:** 2024-06-13

**Authors:** André Berchtold, Noëllie Genre, Francesco Panese

**Affiliations:** Institute of Social Sciences, University of Lausanne, Lausanne, Switzerland; Kore University of Enna: Universita degli Studi di Enna ’Kore’, ITALY

## Abstract

The use of psychotropic drugs among students is well known, but very few studies have been carried out outside North America, and data on Switzerland are particularly scarce. This study investigates the factors that determine the use of drugs and psychotropic substances among students at the University of Lausanne. Our hypotheses were that study pressure could lead to psychotropic drug use; that use could be either regular or experimental; and that users and non-users would have different opinions about the reasons for use and the consequences. Based on a convenience sample (n = 1199) collected by Master’s students from other university students as part of a course given in 2019, our three hypotheses were confirmed. The use of psychotropic drugs is well associated with poorer academic performance. Regarding frequency of use, certain types of psychotropic drugs are used regularly (e.g. antidepressants), while others are used on occasionally (e.g. tranquilizers). Psychotropic substances such as cannabis and cocaine, on the other hand, are mainly used irregularly. Finally, the majority of psychotropic drug users report that they use them as part of their medical treatment, while the majority of non-users suggest that they use them mainly to reduce anxiety and stress in everyday life and at school. Our results show that Switzerland, like other countries, is affected by the phenomenon of psychotropic drug use by students, even outside medical supervision. Accordingly, better information on the negative effects of these substances should then be provided to all university students.

## Introduction

The use of psychotropic drugs by young adults is an important public health issue [1–3]. University students are no exception and are just as concerned as their non-academic peers. However, the bulk of research has been conducted on the North American continent and few studies have been conducted in Europe to date. In the specific case of Switzerland, only a few studies are known: The first used a sample of 6275 students from the Universities of Zurich and Basel, as well as from the ETH Zurich, with the main aim of studying the prevalence of use [4]. Another general study involved the University of Neuchâtel, but only a non-peer-reviewed report was made available [5]. Two other studies focused on more specific questions, one based on a sample of 1765 students at the University of Zurich, comparing users and non-users [6], and one by the University of Geneva focusing on stimulant use [7]. Other research projects collect data on substance use in Switzerland, such as C-SURF for example [8], but do not specifically target university students.

As contexts can vary greatly from one country to another, it is important to have recent data for Switzerland as well. For example, Switzerland is characterized by a very high socioeconomic level, but at the same time the proportion of people with a tertiary education has long lagged behind in international comparisons, and only recently has it risen sharply to over 50% among 30–34 year olds in 2016 [9]. Furthermore, the accessibility of certain psychotropic drugs may differ from one country to country due to different legislation, although the internet greatly facilitates cross-border shopping. University students also represent a special population, as it might be expected that their high level of education would be a protective factor against the use and misuse of psychotropic drugs, but this is not the case [10].

This article focuses primarily on psychotropic drugs, but it is not possible to ignore other psychotropic products as well. To make the distinction, we use the following terminology: The term "psychotropic drugs" includes tranquilizers (anxiolytics), hypnotics, neuroleptics, antidepressants, mood stabilizers, and psychostimulants, and the term "psychotropic substances" includes cannabis, cocaine, amphetamines, hallucinogens, opiates, and other non-medicinal substances. To refer to both categories simultaneously, we will use the term "psychotropic products".

On the basis of literature reviews [11–13], it appears that the use of psychotropic drugs without a doctor’s prescription tends to increase with age. On the other hand, there are inconclusive results regarding gender. While some studies show an association between gender and certain categories of psychotropic drugs, others do not. The use of psychotropic substances such as alcohol and cannabis is positively associated with the use of psychotropic drugs, but causality has not been established. Relatives are an important risk factor for psychotropic drug use if they themselves use psychotropic drugs or engage in other risky behaviors [14, 15]. Similarly, low parental control or more permissive parental attitudes would also be risk factors [16]. Conversely, strong family ties have a protective effect [17], as do religious beliefs. In general, the use of psychotropic drugs without a prescription is more common among people who are prone to risky behavior, and the same is true for people who consider such use to be low risk [18, 19].

The reasons for non-medical use of psychotropic drugs are often multiple, but they can be grouped into three main categories: recreational use, self-medication, and performance enhancement, particularly at school [13, 20]. The use of legal psychotropic substances, especially alcohol, has always been associated with partying [21], whether in a student or non-student setting, but drugs are also used in this context, either for their own effects or to enhance or counteract the effects of other substances [22, 23]. With regard to self-medication, a study of university students in Norway found a significant association between psychological distress and the use of psychotropic drugs [24]. Other studies highlight reasons for use related to sleep disorders and stress [25, 26]. In relation to academic performance, existing studies provide conflicting results. While some studies associate the use of psychotropic drugs with poorer academic performance, others seem to show that the use of certain products, such as stimulants, may be associated with better results [27, 28]. A study by the University of Geneva found that 20% of students had used stimulants in the 6 months prior to the survey, to improve their cognitive performance for exams [7].

The aim of our study was to better understand the use of psychotropic substances in the Swiss university environment and to assess the acceptance of such practices by the students themselves. We therefore addressed the following research question:" Why do university students use psychotropic drugs?" Our work was guided by the following three hypotheses:

1. Study pressure can lead to the use of psychotropic drugs.
2. The use of psychotropic drugs can be regular or experimental.
3. Users and non-users of psychotropic drugs have a different perceptions of the reasons for using such drugs and of the consequences of such use.

The rest of the paper is structured as follows: we begin by describing the data collection and statistical analysis, then we present our results, and finally we conclude with a discussion, returning first to the three hypotheses and then to the other results obtained.

## Methods

### Data

We used a convenience sample of students at the University of Lausanne, Switzerland. A total of n = 1690 students participated in the study, but 424 questionnaires were too incomplete to be used for analyses. In addition, of the remaining 1266 questionnaires, 67 had to be excluded due to significant missing sociodemographic data (these data were collected at the end of the questionnaire). Therefore, the analyses presented here include a total of n = 1199 students.

The questionnaire consisted of 49 questions with a total of 107 items. As many questions depended on the answer to a previous question, most students only had to answer part of the questionnaire. The questionnaire was submitted to the Cantonal Commission for Ethics in Human Research, which issued a decision of non-consideration. The questionnaire was divided into 4 sections: Experiences with the use of drugs and psychotropic substances; perceptions of use and sharing of experiences; quality of life and well-being; and sociodemographic information.

The data were collected between May and June 2019. An invitation email was sent to all bachelor and master students (n = 15’400) at the University of Lausanne with a link to access the online questionnaire. No reminder was sent out and no incentive was given to complete the questionnaire. The software used to carry out the survey was configured to allow only one response per email account, in order to avoid multiple responses, which would have compromised the quality of the data. All the participants were over 18. Participants did not receive any other information about the study apart from the invitation email sent to them. Completing the questionnaire was considered to be consent for the researchers to use the data. The questionnaire was anonymous and, thanks to the large number of students at the University of Lausanne, did not contain any questions that could be used to identify a specific individual, even when several variables were crossed. In addition, the database extracted from the survey software did not contain the IP addresses used by the participants. In the event of psychological difficulties in completing the questionnaire, respondents were referred to the University’s psychotherapeutic counselling service. All questions had to be answered. As incomplete questionnaires were not used in this research, the number of missing data was very small. We therefore decided not to impute missing data.

### Statistical analyses

Respondents were divided into four groups according to their use of psychotropic drugs and/or substances during their time at university: consumers of psychotropic drugs only; consumers of psychotropic substances only; consumers of both psychotropic drugs and substances; no users. We decided not to include substance use prior to entering university entry because the data did not allow us to analyze the reasons for such use.

We first described the main characteristics of the four groups using descriptive statistics (frequencies and percentages for categorical variables, mean and standard deviation for continuous variables). Differences between the groups were systematically tested using the chi-squared test for categorical variables and ANOVA for continuous variables. In a second step, we used logistic regression models to explain why some students used psychotropic drugs and others did not.

All calculations were performed in the statistical environment R [29]. The type I error was set at 5%.

## Results

The total sample comprised 777 Bachelor’s students (305 in their first year), and 422 Master’s students. These 1199 respondents represent 7.8% of all students who were contacted by email to participate in the study. In the sample, 145 (12.1%) students reported having used psychotropic drugs during their time at university, including 58 (4.8%) who also used other psychotropic substances and 87 (7.3%) who did not. In addition, 274 (22.9%) students had used only other psychotropic substances, while 780 (65.1%) students had not used either category of psychotropic substances.

Table 1 describes the main characteristics of these four groups of students. Most variables differed between the 4 groups. Women were more likely to use psychotropic drugs tant men, and consumers were more likely to be over 24 years old. Users were generally in poorer health than non-users and were more likely to have disabilities. They also had more financial problems and were less likely to have a paid job while studying. Overall, users of psychotropic drugs were less satisfied overall with their lives and their relationships with others than non-users. They were also more likely to have problems with their studies. Finally, they were also likely to take risks, such as in their sexual practices or their eating habits. We can also see that people who used psychotropic substances, but not psychotropic drugs, had a profile that was very similar to that of non-users in many aspects. On the other hand, students who used both drugs and psychotropic substances were the group that seemed to be doing the least well off overall.

**Table 1:** Description of the sample. We report the frequencies and the corresponding percentages for categorical variables, and the mean and standard deviation for continuous variables. The last column gives the p-value of a chi-2 test (categorical variables) or an ANOVA (continuous variables) comparing the different user groups.

			User groups					
Variable	Catégories	n	Drugs (n = 87)	Substances (n = 274)	D + S (n = 58)	No-users (n = 780)	Total (n = 1199)	p
**SOCIO-DEMOGRAPHIC**								
**Gender**	Female	806	75.9%	53.6%	51.7%	72.2%	67.2%	<0.001
	Male	385	23.0%	45.3%	46.6%	27.4%	32.1%	
	Other	8	1.1%	1.1%	1.7%	0.4%	0.7%	
**Age**	< = 24	915	57.5%	78.5%	69.0%	78.2%	76.3%	<0.001
	>24	284	42.5%	21.5%	31.0%	21.8%	23.7%	
**Health status**	Good	1102	78.2%	95.6%	74.1%	93.5%	91.9%	<0.001
	Poor	97	21.8%	4.4%	25.9%	6.5%	8.1%	
**Handicap**	Yes	53	17.2%	3.6%	13.8%	2.6%	4.4%	<0.001
	No	1146	82.8%	96.4%	86.2%	97.4%	95.6%	
**Study level**	Bachelor, 1^st^ y.	305	19.5%	23.7%	27.6%	26.5%	25.4%	0.659
	Bachelor, 2–3 y.	472	41.4%	38.0%	36.2%	39.9%	39.4%	
	Master	422	39.1%	38.3%	36.2%	33.6%	35.2%	
**Financial status**	Good	938	69.0%	76.3%	62.1%	81.2%	78.2%	<0.001
	Poor	261	31.0%	23.7%	37.9%	18.8%	21.8%	
**Scholarship**	Yes	138	13.8%	10.9%	12.1%	11.4%	11.5%	0.907
	No	1061	86.2%	89.1%	87.9%	88.6%	88.5%	
**Paid job**	Yes	675	58.6%	65.7%	56.9%	52.7%	56.3%	0.003
	No	524	41.4%	34.3%	43.1%	47.3%	43.7%	
**QUALITY OF LIFE**								
**Overall satisfied with my life**	Agree	958	69.0%	83.2%	53.4%	81.9%	79.9%	<0.001
	Disagree/no opinion	241	31.0%	16.8%	46.6%	18.1%	20.1%	
**Few reasons to be proud of me**	Agree	177	14.9%	11.7%	32.8%	14.5%	14.8%	<0.001
	Disagree/no opinion	1022	85.1%	88.3%	67.2%	85.5%	85.2%	
**Fulfilled in my studies**	Agree	805	63.2%	70.4%	43.1%	68.2%	67.1%	<0.001
	Disagree/no opinion	394	36.8%	29.6%	56.9%	31.8%	32.9%	
**Supported by my entourage**	Agree	1031	79.3%	86.1%	74.1%	87.6%	86.0%	0.009
	Disagree/no opinion	168	20.7%	13.9%	25.9%	12.4%	14.0%	
**Satisfied with my social relationships**	Agree	946	64.4%	83.6%	56.9%	80.5%	78.9%	<0.001
	Disagree/no opinion	253	35.6%	16.4%	43.1%	19.5%	21.1%	
**I can manage my life the way I want**	Agree	865	62.1%	74.5%	55.2%	73.7%	72.1%	0.002
	Disagree/no opinion	334	37.9%	25.5%	44.8%	26.3%	27.9%	
**STUDIES**								
**Feel overwhelmed by the level required**	Yes	347	39.1%	24.1%	37.9%	28.8%	28.9%	0.021
	No/no opinion	852	60.9%	75.9%	62.1%	71.2%	71.1%	
**Academic failures**	Yes	311	44.8%	22.6%	44.8%	23.6%	25.9%	<0.001
	No/no opinion	888	55.2%	77.4%	55.2%	76.4%	74.1%	
**Demotivated by studies**	Yes	318	29.9%	26.6%	50.0%	24.4%	26.5%	<0.001
	No/no opinion	881	70.1%	73.4%	50.0%	75.6%	73.5%	
**Problems of integration**	Yes	38	2.3%	1.5%	8.6%	3.5%	3.2%	0.034
	No/no opinion	1161	97.7%	98.5%	91.4%	96.5%	96.8%	
**RISK-TAKING**								
**Unsafe sexual practices**	Yes	112	9.2%	13.5%	31.0%	6.3%	9.3%	<0.001
	No	1087	90.8%	86.5%	69.0%	93.7%	90.7%	
**Social relationships emotionally burdensome**	Yes	310	40.2%	29.9%	58.6%	20.4%	25.9%	<0.001
	No	889	59.8%	70.1%	41.4%	79.6%	74.1%	
**Endangerment in a sporting activity**	Yes	40	4.6%	6.2%	10.3%	1.7%	3.3%	<0.001
	No	1159	95.4%	93.8%	89.7%	98.3%	96.7%	
**Over-, under- or misfeeding**	Yes	282	25.3%	23.7%	41.4%	21.9%	23.5%	0.009
	No	917	74.7%	76.3%	58.6%	78.1%	76.5%	
**Too much time on the smartphone/hyperconnectivity**	Yes	572	49.4%	50.0%	62.1%	45.6%	47.7%	0.078
	No	627	50.6%	50.0%	37.9%	54.4%	52.3%	
**DRUGS AND SUBSTANCES**								
**Age at first use of psychotropic drugs**		145	19.6 (4.5)	-	19.2 (3.2)	-	19.4 (4.1)	0.583
**Age at first use of psychotropic substances**		332	-	17.1 (2.8)	16.4 (2.5)	-	17.0 (2.8)	0.079

The next two tables show which psychotropic products were used by the students. Table 2 deals with psychotropic drugs and Table 3 with psychotropic substances. In both cases, we show the percentage of students who had already used each product since they entered university, as well as the percentage of students who used these products regularly, i.e. at least several times a week. Among psychotropic drugs, tranquilizers were used by the largest number of students, followed by psychostimulants and antidepressants. However, in terms of regular use, antidepressants were used most frequently, which is not surprising given that antidepressants need to be taken every day in order to be effective and well tolerated by the body, followed by psychostimulants. There was no significant difference in use between the group of people who used only psychotropic drugs and those who used both drugs and psychotropic substances. Among psychotropic substances, cannabis was by far the most commonly used (86.1% of all users of psychotropic products), followed by amphetamines and hallucinogens. Cannabis was also the most regularly used substance (16.6% of users). On the other hand, most substances (cocaine, amphetamines, hallucinogens, opiates) although used by some students, were never or hardly ever used regularly probably because of their recreational purpose, but also because of the pharmacological way in which they have to be taken. It should also be note that cocaine, amphetamines and opiates were used more frequently by students who also used psychotropic drugs than by students who only used psychotropic substances.

**Table 2:** Psychotropic drug use. The “total users” line represents all respondents who have used drugs since entering university and the “regular users” line indicates those who used drugs several times a week or daily. The last column gives the p-value of a chi-2 test comparing the group of drugs only users with the group of students using both drugs and substances.

		User groups			
		Drugs (n = 87)	D + S (n = 58)	Total (n = 145)	p
**Tranquilizers**	**Total users n (%)**	48 (55.2%)	34 (58.6%)	82 (56.6%)	0.811
	**Regular users n (%)**	8 (9.2%)	5 (8.6%)	13 (9.0%)	1.000
**Hypnotics**	**Total users n (%)**	17 (19.5%)	14 (24.1%)	31 (21.4%)	0.649
	**Regular users n (%)**	3 (3.4%)	5 (8.6%)	8 (5.5%)	0.334
**Neuroleptics**	**Total users n (%)**	7 (8.0%)	4 (6.9%)	11 (7.6%)	1.000
	**Regular users n (%)**	3 (3.4%)	1 (1.7%)	4 (2.8%)	0.918
**Antidepressants**	**Total users n (%)**	28 (32.2%)	22 (37.9%)	50 (34.5%)	0.593
	**Regular users n (%)**	22 (25.3%)	14 (24.1%)	36 (24.8%)	1.000
**Mood stabilizers**	**Total users n (%)**	5 (5.7%)	3 (5.2%)	8 (5.5%)	1.000
	**Regular users n (%)**	3 (3.4%)	1 (1.7%)	4 (2.8%)	0.918
**Psychostimulants**	**Total users n (%)**	26 (29.9%)	26 (44.8%)	52 (35.9%)	0.097
	**Regular users n (%)**	11 (12.6%)	10 (17.2%)	21 (14.5%)	0.596

**Table 3:** Psychotropic substance use. The first line represents all respondents who have used substances since entering university and the second line indicates those who used substances several times a week or daily. The last column gives the p-value of a chi-2 test comparing the group of substances only users with the group of students using both drugs and substances.

		User groups			
		Substances (n = 274)	D + S (n = 58)	Total (n = 332)	p
**Cannabis**	**Total users n (%)**	239 (87.2%)	47 (81.0%)	286 (86.1%)	0.303
	**Regular users n (%)**	45 (16.4%)	10 (17.2%)	55 (16.6%)	1.000
**Cocaine**	**Total users n (%)**	41 (15.0%)	19 (32.8%)	60 (18.1%)	0.003
	**Regular users n (%)**	0 (0.0%)	0 (0.0%)	0 (0.0%)	-
**Amphetamines**	**Total users n (%)**	83 (30.3%)	31 (53.4%)	114 (34.3%)	0.001
	**Regular users n (%)**	1 (0.4%)	0 (0.0%)	1 (0.3%)	1.000
**Hallucinogens**	**Total users n (%)**	57 (20.8%)	14 (24.1%)	71 (21.4%)	0.699
	**Regular users n (%)**	0 (0.0%)	0 (0.0%)	0 (0.0%)	-
**Opiates**	**Total users n (%)**	3 (1.1%)	6 (10.3%)	9 (2.7%)	<0.001
	**Regular users n (%)**	0 (0.0%)	3 (5.2%)	3 (0.9%)	0.003
**Other non-medicinal substances**	**Total users n (%)**	29 (10.6%)	13 (22.4%)	42 (12.7%)	0.025
	**Regular users n (%)**	6 (2.2%)	1 (1.7%)	7 (2.1%)	1.000

Table 4 provides information on the source of psychotropic drugs used by students. Almost three quarters of the students reported that these drugs were part of a medical treatment, but other sources were also frequently mentioned, particularly drugs provided by friends (24.1%) or by family members (21.4%). On the other hand, only 4.1% of users reported buying drugs over the internet.

**Table 4:** Origin of the psychotropic drugs (multiple answers possible), n = 145.

	n	%
**As part of my medically monitored treatment**	106	73.1
**Given by a family member**	31	21.4
**Given by a friend**	35	24.1
**Given by a student**	11	7.6
**Purchased from someone I know**	8	5.5
**Purchased from someone I don’t know**	8	5.5
**Purchased on the internet**	6	4.1
**Purchased in a pharmacy / store**	18	12.4
**Other**	3	2.1

Table 5 shows the effects perceived by users of psychotropic drug. Overall, these effects were in line with their expectations, and users were not very worried about their consumptions. In detail, the effects with which the respondents most strongly agreed were feeling more relaxed in social situations (46.2%), falling asleep more easily (58.6%), feeling more competent and efficient (42.8%), being able to concentrate better (53.1%) and having greater self-confidence (40.6%). The most common negative effects were loss of appetite, heart palpitations, nervousness and feelings of guilt, but these negative effects always affected less than 30% of the consumers.

**Table 5:** Self-reported effects of the consumption of psychotropic drugs.

	Strongly agree	Agree	No opinion	Disagree	Strongly disagree
**I feel more serene, calm**	9 (6.2%)	23 (15.9%)	28 (19.3%)	32 (22.1%)	53 (36.6%)
**I feel more relaxed in social situations**	33 (22.8%)	34 (23.4%)	42 (29.0%)	23 (15.9%)	13 (9.0%)
**I can fall asleep more easily**	48 (33.1%)	37 (25.5%)	22 (15.2%)	23 (15.9%)	15 (10.3%)
**I feel asleep**	25 (17.2%)	39 (26.9%)	22 (15.2%)	36 (24.8%)	23 (15.9%)
**I feel more competent, performant**	20 (13.8%)	42 (29.0%)	34 (23.5%)	30 (20.7%)	19 (13.1%)
**I can concentrate better**	28 (19.3%)	49 (33.8%)	25 (17.2%)	24 (16.6%)	19 (13.1%)
**I feel more confident**	19 (13.1%)	40 (27.5%)	46 (31.7%)	28 (19.3%)	12 (8.3%)
**I lose my appetite**	12 (8.3%)	31 (21.4%)	34 (23.4%)	30 (20.7%)	38 (26.2%)
**I have palpitations**	9 (6.2%)	33 (22.8%)	24 (16.6%)	28 (19.3%)	51 (35.2%)
**I have nausea**	3 (2.1%)	31 (21.4%)	25 (17.2%)	31 (21.4%)	55 (37.9%)
**I feel nervous**	3 (2.1%)	29 (20.0%)	31 (21.4%)	33 (22.8%)	49 (33.8%)
**I feel angry**	3 (2.1%)	13 (9.0%)	31 (21.4%)	37 (25.5%)	61 (42.1%)
**I feel aggressive**	4 (2.8%)	11 (7.6%)	27 (18.6%)	40 (27.6%)	63 (43.4%)
**I feel guilty**	9 (6.2%)	23 (15.9%)	28 (19.3%)	32 (22.1%)	53 (36.6%)
**The effects felt corresponded to my expectations**	36 (24.8%)	85 (58.6%)	8 (5.5%)	16 (11.0%)	0 (0.0%)
**I’m worried about taking psychotropic drugs**	4 (2.8%)	26 (17.9%)	19 (13.1%)	47 (32.4%)	49 (33.8%)

Table 6 shows that although the majority of psychoactive drug users had discussed their use with others, most of these discussions were not with fellow students. Instead, the main people involved were friends (although it is likely that some of these were also students), family members and health care professionals. While some users felt negatively judged when they talked about their use of psychotropic drugs, an equal number did not experience such negative judgement.

**Table 6:** How do psychotropic drug users talked about their consumption with other people.

	Categories	n	%
**Do you inform people about your use of psychotropic**	Yes	113	77.9
**drugs? (n = 145)**	No	32	22.1
**Do you exchange information about psychotropic drugs**	Yes	67	46.2
**with other students? (n = 145)**	No	78	53.8
**Overall, talking about it with other students:**	Generally discourages me from using it	9	13.4
**(if Yes at the previous question)**	Generally encourages me to use it	2	3.0
**(n = 67, 2 missing data)**	Has no influence	56	83.6
**Who do you talk to about your psychotropic drug use?**	Family	93	64.1
**(n = 113, if Yes at the first question, multiple answers possible)**	Friends	99	68.3
	Other students	35	24.1
	Health professionals	57	39.3
	University professionals	5	3.4
	Other people	7	4.8
**I feel negatively judged when I tell people about my use**	Fully agree	7	6.2
**of psychotropic drugs**	Agree	33	29.2
**(n = 113, if Yes at the first question)**	No opinion	23	20.4
	Disagree	38	33.6
	Fully disagree	12	10.6

Table 7 compares the opinions of users and non-users of psychotropic drugs on the reasons for taking such drugs. It appears that the opinions of the two groups are very significantly different. In particular, users were much more likely than non-users to say that they were prescribed these drugs as part of a medical treatment. On the other hand, the latter very often imagined that users were trying to reduce their stress or anxiety, whether in everyday life or in relation to their studies, to improve their academic performance or simply out of curiosity. The only point on which the two groups of respondents agreed was on the use of substances to cope with anxiety about the uncertainties of the future. In this context, Table 8 provides information on the use of psychotropic drugs outside of a doctor’s prescription. Again, the opinions of the two groups differed significantly on all the questions asked, with non-users being much more worried than users.

**Table 7:** Reasons for using psychotropic drugs (multiple answers possible). Comparison of the point of view of users and non-users of such drugs during university time. The last column gives the p-value of a t-test comparing the two groups.

Reasons to consume psychotropic drugs	Users (n = 145)	Non-users (n = 1054)	p
**On prescription from a doctor as part of a treatment**	71.0%	51.3%	<0.001
**Following a self-diagnosis**	9.0%	18.7%	<0.001
**To reduce anxiety or stress related to daily life**	45.5%	80.4%	<0.001
**To manage the stress level before a particular academic event (exam, test, etc.)**	35.9%	76.0%	<0.001
**To improve academic performance (concentration, attention, memory, etc.)**	29.7%	65.8%	<0.001
**To improve the quality of sleep**	24.8%	42.9%	<0.001
**Out of curiosity or for recreational use**	15.2%	63.1%	<0.001
**To facilitate relationships or social integration**	11.0%	30.9%	<0.001
**To manage fears about an uncertain future**	19.3%	23.7%	0.215

**Table 8:** Perception of students taking psychotropic drugs outside of medical treatment, comparing users and non-users. We provide the percentage of respondents who "strongly agree" or "agree" with each statement. The last column gives the p-value of a chi-2 test comparing the two groups.

Perception of psychotropic drug users	Users (n = 145)	Non-users (n = 1054)	p
**They put themselves in danger**	54.5%	74.9%	<0.001
**It worries me**	44.8%	61.1%	<0.001
**It bothers me**	11.7%	25.0%	<0.001
**The fact that it does them good justifies it**	41.4%	17.6%	<0.001
**There is a need to take these drugs**	57.9%	32.6%	<0.001
**If it increases cognitive ability, it is unfair to students who do not take it**	20.0%	34.2%	<0.001

Table 9 presents three regression models to explain psychotropic drug use among university students. The first model uses only academic explanatory factors, the second model adds health-related factors, and the third model adds socio-demographic factors. Looking at the final model, we find that despite the addition of other explanatory factors, individuals who had experienced academic failure had a more than doubled risk of using psychotropic drugs (OR = 2.13). Among the other factors, the most significant was having a disability (OR = 5.09), followed by poor health (OR = 2.49). Emotionally distressing social relationships (OR = 2.30) and the use of psychotropic substances (OR = 1.60) were also risk factors, as was being older than 24 years (OR = 2.18).

**Table 9:** Logistic regression models for the consumption of psychotropic drugs vs non-consumption (reference category).

		Model 1		Model 2		Model 3	
Variable	Categories	OR	p	OR	p	OR	p
**Feel overwhelmed by the level**	Yes	1.24	0.265	1.26	0.268	1.38	0.132
**required**	No/no opinion (ref)						
**Academic failures**	Yes	**2.47**	**<0.001**	**2.14**	**<0.001**	**2.13**	**<0.001**
	No/no opinion (ref)						
**Demotivated by studies**	Yes	**1.52**	**0.043**	1.30	0.234	1.28	0.277
	No/no opinion (ref)						
**Problems of integration**	Yes	1.31	0.531	1.02	0.959	0.84	0.723
	No/no opinion (ref)						
**Fulfilled in my studies**	Disagree/no opinion	1.35	0.137	1.21	0.386	1.24	0.342
	Agree (ref)						
**Health status**	Poor			**2.57**	**<0.001**	**2.49**	**0.001**
	Good (ref)						
**Handicap**	Yes			**5.43**	**<0.001**	**5.09**	**<0.001**
	No (ref)						
**Over-, under- or misfeeding**	Yes			0.91	0.686	0.85	0.483
	No (ref)						
**Psychotropic substance use**	Yes			**1.57**	**0.028**	**1.60**	**0.027**
	No (ref)						
**Unsafe sexual practices**	Yes			1.44	0.198	1.46	0.188
	No (ref)						
**Social relationships**	Yes			**2.32**	**<0.001**	**2.30**	**<0.001**
**emotionally burdensome**	No (ref)						
**Endangerment in**	Yes			1.76	0.176	1.86	0.145
**a sporting activity**	No (ref)						
**Gender**	Male					1.11	0.648
	Other					0.64	0.658
	Female (ref)						
**Age**	>24					**2.18**	**<0.001**
	< = 24 (ref)						
**Financial status**	Poor					1.16	0.504
	Good (ref)						
**Paid job**	Yes					0.90	0.598
	No (ref)						

## Discussion

The analyses confirmed our three working hypotheses. First, academic pressure was indeed related to psychotropic drug use. The descriptive results in Table 1 showed that psychotropic drug users had more negative feelings about how well their studies were going than non-users. Moreover, even after controlling for other risk factors (Table 9), academic failure remained a highly significant factor. This findings is in line with what has already been established in the literature, whether at high school [30, 31] or university level [32]. This implies that, in addition to the usual prevention campaigns, additional measures should be proposed in order to limit the use of psychotropic drugs, both by better explaining their harmful consequences and by improving the well-being of student so that they no longer need to resort to them.

Our second hypothesis concerned the frequency of psychotropic drug use, namely regular or experimental. The results in Table 2 showed that for all the psychotropic drugs considered, use could vary from one student to another with some using drugs regularly (antidepressants, and psychostimulants in particular) and others much more occasionally (tranquilizers and hypnotics in particular). This contrasts with the use of psychotropic substances, which was mainly irregular (Table 3). This regular use confirmed the fact that some students were using psychotropic drugs for treatment purposes, as most classes of psychotropic drugs require daily dosing rather than sporadic use, indicating a medical regimen rather than recreational intent. In fact, 73.1% of psychotropic drug users reported that they had used them as part of a medical treatment. It is therefore highly plausible that the use of such drugs was often initiated by a prescription based on a medical diagnosis. However, steps must be taken to ensure that drug use that begins under medical supervision does not continue unchecked. This is in line with the findings of a literature review which suggests that one in three students prescribed a psychotropic drug subsequently diverts its use to other purposes [11].

Finally, our third hypothesis examined the differences in perceptions between users and non-users of psychoactive drugs. The results in Table 7 tended to show that the reasons for using psychotropic drugs as imagined by non-users differed considerably from those given by users. As mentioned above, the main reason given by users was that it was part of a medical treatment. None of the other reasons given reached 50% agreement, the most common being the need to reduce anxiety and stress in everyday life (45.5%). Conversely, non-consumers believed that this last reason was true for 80.4% of consumers, followed by study stress related (76.0%) and the desire to improve academic performance (65.8%). Although it is possible that the users may have consciously or unconsciously concealed some of their motivations for using, the systematic aspect of the differences recorded between users and non-users cannot be attributed to chance alone, and we can hypothesize that a certain amount of fantasy surrounds the use of these drugs. Directly related to this, our analyses also showed that non-users were more concerned about the possible negative effects (e.g. side effects, dependence, …) of psychotropic drug use than users themselves (Table 8). This difference may be due to various factors, such as a lack of knowledge about these drugs and their medical follow-up by the non-users, but also to a more or less conscious minimization of the negative effects by the users, who preferred to keep only the positive effects of these medications, perhaps in order to be able to continue their treatment, whether prescribed by a doctor or not. Our findings can be linked to a study showing that strategies for controlling one’s own drug use, and perceptions of such strategies, can vary widely between users and non-users [33]. We can also cite another study showing that the general population tends to overestimate the prevalence of substance use and underestimate the chances of stopping it [34]. It follows that it is necessary to strike a fair balance between the a priori attitudes that may exist towards users of psychotropic products and the reality and conditions of their use.

Psychotropic products include not only drugs but also illicit substances such as cannabis, cocaine or hallucinogens. As the use of such substances is more common than the use of psychotropic drugs, and as a significant proportion of the drug users in our sample also used substances, it was logical to include them in our analyses as well. The results showed that, unlike drugs, substances were almost never used on a regular basis (at least several times a week), suggesting essentially recreational rather than therapeutic use. However, we found that the use of cocaine, amphetamines and opiates was more frequent, although not regular, among users of psychotropic drugs than among non-users of these drugs. We can hypothesize either that the use of psychotropic drugs tends to normalize the use of psychotropic drugs in general, or that, in the absence of drugs, there is a substitution effect with other substances. On the other hand, the comparison between substance users, psychotropic drug users and non-users showed that substance users who did not use drugs had very similar characteristics to non-users. In particular, their health was generally very good, they were satisfied with their lives and they had few problems with their studies. This is consisted with what has previously been found in relation to cannabis [35]. The only real difference between substance users and non-users was that substance users took more risks than non-users. In contrast, students who used psychotropic drugs, whether or not in combination with psychotropic substances, generally did worse than the other two groups of students.

The proportions of psychotropic substance and drug users found in our study are similar to those observed in a study conducted in France among pharmacy students, although in the latter case only the use in the previous 3 months was considered [36]. In contrast, another study conducted in Brazil among pharmacy students found a much higher percentage of over-the-counter use of psychotropic drugs (79.2%) [37]. However, these were students attending evening classes and were on average older than the respondents in our own sample, and it is known that psychotropic drug abuse tends to increase with age [38, 39]. Compared with the total sample, women were more likely to be non-users of psychotropic substances or users of psychotropic drugs only, whereas men were more likely to be users of psychotropic substances, in combination or not with psychotropic drugs.

Although 71% of psychotropic substance users reported that at least some of these drugs were taken as part of a medical treatment, many other reasons for self-medication were reported by respondents. This must be related to the means of obtaining these drugs. Again, over 70% of consumers reported legal supply as part of a medical treatment, but many other sources were also reported, including family, friends, and purchases from pharmacies and the Internet. This is consistent with the existing literature, which highlights the variety of methods used to obtain psychotropic drugs, including theft [12]. On the other hand, it may be at odds with what De Souza et al. found with American students, for whom access to psychotropic drugs seemed to come mainly from friends who were already using them, rather than from health professionals [40]. Moreover, even if many psychotropic drugs are used as part of a medical treatment, this does not eliminate all risks. In fact, a study conducted in the United States showed that even within a treatment regimen, the dosage was not always adhered to, with frequent cases of over- or under-dosing [41], as is also the case, for example, in people with epilepsy who are being treated [42].

The context of psychotropic use may also differ between drugs or substances. As reported by McCabe et al., psychotropic substances are more likely used in a festive context in the presence of others, whereas psychotropic drugs are much more likely to be used when people are alone [43]. This is partly reflected in our study, since recreational use or use to facilitate social integration of psychotropic drugs was reported by only a minority of users, contrary to the opinion of non-users. When users of psychoactive drugs discussed their use with others, it was more likely to be with health professionals, friends or family members than with other students. This may be due to the private nature of drug use in the context of illness and treatment, but it may also be due to the fact that discussions with other students generally did not change the users’ own opinions (Table 6). The fear of being judged negatively, although not felt by everyone, may also have played a role.

This article presents data on the use of psychotropic drugs by students at the University of Lausanne. It is original because, in addition to the consumption itself, it also provides information on the feelings of users and non-users regarding the fact of consumption. The main limitation of this research is that it is based on a convenience sample that does not claim to represent all students at the University of Lausanne, nor university students in general. This means that it is not possible to generalize the results directly, either to all students at the University of Lausanne or to other populations. This must be clearly stated when referring to our results. In addition, the cross-sectional nature of the study makes it impossible to establish a causal link. Another important limitation is directly related to the choice of questions included in the study. The literature has shown that there are strong associations between the (poly-)use of illicit and licit substances, but we did not have information on the use of alcohol and tobacco products by the respondents in our survey. Our study is therefore unfortunately incomplete in this respect. In order to address the above limitations, a new study with a more comprehensive questionnaire and longitudinal data collection should be conducted. However, despite these various limitations, our analyses allow us to highlight interesting differences between users and non-users of psychotropic drugs, and to compare the opinions of these two groups.

Our results show that Switzerland, like other countries, is affected by the phenomenon of psychotropic drug use by students outside medical treatment. Belonging to the most educated part of the population is not a protective factor, and specific research into the causes of substance use among students should be explored, for example through qualitative research, to find out more about the processes involved for this specific population (i.e. academic pressure, our first hypothesis, and the context of use and frequency, our second hypothesis). Mamat et al. [11] have clearly shown that the use of illicit psychotropic drugs is increasing worldwide, and although we lack specific data for Switzerland, other recent studies confirm the magnitude of the problem [24, 44–46]. This steady increase is an indicator that the problem is far from being under control. In Switzerland, we can speculate that this may be related to a lack of coordination between health care actors. For example, we have no record of all the prevention campaigns that have been carried out over the last 20 years, although we know that many campaigns have been carried out at city, cantonal and federal level, targeting various legal and illegal substances. The lack of such a register makes it difficult to assess the relative merits and effectiveness of different types of prevention campaigns.

In line with public health recommendations [47], health professionals should also be better trained to recognize problematic situations in the student population in order to prevent drug and substance abuse from getting out of control. Furthermore, as shown by the results related to our third hypothesis (perception of users and non-users), better information could be provided to students when they enter university to remind them of the dangers of taking psychotropic products outside medical supervision. In this regard, we can hypothesize that the fact that more and more people are receiving prescriptions for psychotropic drugs [48, 49] could exacerbate illegal use by allowing greater access to drugs and greater familiarity with the whole family of products. Therefore, the difference between legal and illegal use in terms of consequences should be emphasized. Finally, students should be better informed about the health services to which they should turn if they have a problem.

## Supporting information

S1 File Questionnaire. Final version of the questionnaire.
(PDF)
