# Supplementary material for: Uses of psychotropic drugs by university students in Switzerland
Source: PLoS One. 2024 Jun 13;19(6):e0305392. doi: 10.1371/journal.pone.0305392 (PMC11175452; doi:10.1371/journal.pone.0305392)
Supplement: S1 File — Final version of the questionnaire. (PDF) [file pone.0305392.s001.pdf]

Enquête auprès de la communauté des étudiant·e·s de l'Université de Lausanne sur la prise de médicaments et de substances psychotropes

L'objectif de ce questionnaire est de mieux connaître la vie des étudiant.e.s de l'UNIL afin d'identifier des points à améliorer. Parce qu'il est impossible de rendre compte de toute sa complexité, nous tentons d'en saisir des dimensions importantes à partir d'une thématique, d'un analyseur : *la prise de médicaments et de substances psychotropes dans la population étudiante*.

On appelle psychotrope, une substance chimique d'origine naturelle ou artificielle, [...] qui est suceptible de modifier l'activité mentale (Delay, 1967)

Ce questionnaire est bien-sûr entièrement anonyme. Son but est de recueillir votre expérience et votre avis sur cette question de manière ouverte et sans aucun jugement.

Cette enquête est menée par une classe de Master en sciences sociales de la santé auprès de tous les étudiant.e.s de l'UNIL avec l'aval de sa Direction. Nous sommes donc des étudiant·e·s qui nous adressons à nos pairs.

Remplir ce questionnaire prendra environ 20 minutes qui permettront de connaître et de faire reconnaitre votre expérience sur des questions importantes qui nous concernent toutes et tous en premier lieu.

Si en répondant à ce questionnaire vous estimez que vous présentez des difficultés psychologiques, ne restez pas seul/e. Vous pouvez obtenir de l'aide après des services suivants :

En cas d'urgence, vous pouvez en tout temps (24h/24 et 7/7 jours) téléphoner au numéro 0848 133 133 (Centrale téléphonique des médecins de garde) et vous serez orientés au mieux.

Hors urgence, vous pouvez prendre rendez-vous par mail à la Consultation psychothérapeutique pour étudiants de l'UNIL : consult.psy-unil@chuv.ch

La première consultation est gratuite et la confidentialité est garantie.

Il y a 49 questions dans ce questionnaire

PARTIE A : EXPERIENCES DE PRISES DE MEDICAMENTS ET SUBSTANCES PSYCHOTROPES

Dans cette partie vous allez pouvoir faire part de vos expériences liées à la prise de médicaments et substances psychotropes.

Pour rappel, on appelle psychotrope, une substance chimique d'origine naturelle ou artificielle, [...] qui est suceptible de modifier l'activité mentale (Delay, 1967)

[]Faites-vous ou avez-vous fait l'expérience de prises de médicaments psychotropes?

*

Veuillez sélectionner une seule des propositions suivantes :

- ○ Oui
- ○ Non

[]Quels sont les médicaments psychotropes dont vous faites/avez fait l'expérience ? *

Répondre à cette question seulement si les conditions suivantes sont réunies :
La réponse était 'Oui' à la question '1 [Conso1]' (Faites-vous ou avez-vous fait l'expérience de prises de médicaments psychotropes?)

Choisissez la réponse appropriée pour chaque élément :

	ne me concerne pas	au cours de ma vie	au cours de mon cursus universitaire	au cours de la dernière année
Tranquillisants (anxiolytiques): Xanax®, Lexotanil®, Lexomil®, Urbanyl®, Rivotril®, Tranxillium®, Valium®, Temesta®, Anxiolit®, Seresta®, Démétrin®	○	○	○	○
Hypnotiques (somnifères): Dalmadorm®, Dormicum®, Stilnox®, Imovane®, Sonata®, Buspar®, Atarax®	○	○	○	○
Neuroleptiques (antipsychotiques): Nozinan®, Truxal®, Entumine®, Clopixol®, Fluanxol®, Dipipéron®, Haldol®, Halopéridol®, Abilify®, Leponex®, Risperdal®, Zyprexa®, Seroquel®, Zeldrox®, Latuda®	○	○	○	○
Antidépresseurs (thymoanaleptiques): Anafranil®, Tolvon®, Saroten®, Surmontil®, Trittico®, Prozac®, Cymbalta®, Fluctine®, Zoloft®, Gladem®, Floxyfral®, Flox-ex®, Deroxat®, Seropam®, Cipralex®, Wellbutrin®, Zyban®, Efexor®, Effexor®, Remeron®	○	○	○	○
Stabilisateurs de l'humeur (thymorégulateur): Lyrica®, Neurontin®, Topamax®, Tégrétol®, Dépamide®, Dépakine chrono®, Lamictal®, Lithiofor®	○	○	○	○
Psychostimulants: Ritaline®, Focalin®, Concerta®, Modasomil®	○	○	○	○

[]A quelle fréquence? *

Répondre à cette question seulement si les conditions suivantes sont réunies :
La réponse était 'Oui' à la question '1 [Conso1]' (Faites-vous ou avez-vous fait l’expérience de prises de médicaments psychotropes?)

Choisissez la réponse appropriée pour chaque élément :

	Jamais	Quotidiennement	Régulièrement (plusieurs fois par semaine)	Ponctuellement (liée à un/des événement(s) ou moment(s) spécifique(s)	Rarement (quelques fois durant l’année)	Une seule fois lors de situation(s)/ événement(s) spécifique(s)
Tranquillisants (anxiolytiques)	○	○	○	○	○	○
Hypnotiques (somnifères)	○	○	○	○	○	○
Neuroleptiques (antipsychotiques)	○	○	○	○	○	○
Antidépresseurs (thymoanaleptiques)	○	○	○	○	○	○
Stabilisateurs de l’humeur (thymorégulateurs)	○	○	○	○	○	○
Psychostimulants	○	○	○	○	○	○

[]A quel âge avez-vous pris pour la première fois un de ces médicament(s) psychotrope(s) ? *

Répondre à cette question seulement si les conditions suivantes sont réunies :
La réponse était 'Oui' à la question '1 [Conso1]' (Faites-vous ou avez-vous fait l’expérience de prises de médicaments psychotropes?)

Seuls des nombres peuvent être entrés dans ce champ.

Veuillez écrire votre réponse ici :

[]Faites-vous ou avez-vous déjà fait l’expérience de prises de substances psychotropes (drogues)?

*

Veuillez sélectionner une seule des propositions suivantes :

- ○ Oui
- ○ Non

[]Quelles sont les substances psychotropes dont vous faites/avez fait l’expérience ? *

Répondre à cette question seulement si les conditions suivantes sont réunies :
La réponse était 'Oui' à la question '5 [Consosub]' (Faites-vous ou avez-vous déjà fait l’expérience de prises de substances psychotropes (drogues)?)

Choisissez la réponse appropriée pour chaque élément :

	ne me concerne pas	au cours de ma vie	au cours de mon cursus universitaire	au cours de la dernière année
Cannabis	○	○	○	○
Cocaïne	○	○	○	○
Amphétamines (Ecstasy, MDMA, « speed », méthamphétamine, etc.)	○	○	○	○
Hallucinogènes (LSD, champignons, peyotls, etc.)	○	○	○	○
Opiacées (héroïne, opium, etc.)	○	○	○	○
Autres drogues	○	○	○	○

[]A quelle fréquence? *

Répondre à cette question seulement si les conditions suivantes sont réunies :
La réponse était 'Oui' à la question '5 [Consosub]' (Faites-vous ou avez-vous déjà fait l’expérience de prises de substances psychotropes (drogues)?)

Choisissez la réponse appropriée pour chaque élément :

	Jamais	Quotidiennement	Régulièrement (plusieurs fois par semaine)	Ponctuellement (liée à un/des événement(s) ou moment(s) spécifique(s)	Rarement (quelques fois durant l’année)	Une seule fois lors de situation(s)/ événement(s) spécifique(s)
Cannabis	○	○	○	○	○	○
Cocaïne	○	○	○	○	○	○
Amphétamines (Ecstasy, MDMA, « speed », méthamphétamine, etc.)	○	○	○	○	○	○
Hallucinogène (LSD, champignons, peyotls, etc.)	○	○	○	○	○	○
Opiacées (héroïne, opium, etc.)	○	○	○	○	○	○
Autres	○	○	○	○	○	○

[]A quel âge avez-vous pris pour la première fois une de ces substance(s) psychotrope(s)? *

Répondre à cette question seulement si les conditions suivantes sont réunies :
La réponse était 'Oui' à la question '5 [Consosub]' (Faites-vous ou avez-vous déjà fait l’expérience de prises de substances psychotropes (drogues)?)

Seuls des nombres peuvent être entrés dans ce champ.

Veuillez écrire votre réponse ici :

[ ]

PARTIE A(2) : EXPERIENCES DE PRISES DE MEDICAMENTS ET SUBSTANCES PSYCHOTROPES

[]Vous est-il arrivé de prendre conjointement et délibérément plusieurs de ces médicaments ou substances psychotropes? *

Répondre à cette question seulement si les conditions suivantes sont réunies :
La réponse était 'Oui' à la question '1 [Conso1]' (Faites-vous ou avez-vous fait l'expérience de prises de médicaments psychotropes?)

Veuillez sélectionner une seule des propositions suivantes :

- ○ Oui
- ○ Non

[]Lesquels? *

Répondre à cette question seulement si les conditions suivantes sont réunies :
La réponse était 'Oui' à la question '9 [Conso5]' (Vous est-il arrivé de prendre conjointement et délibérément plusieurs de ces médicaments ou substances psychotropes?)

Veuillez choisir toutes les réponses qui conviennent :

- ☐ Médicament(s) psychotrope(s) dans le cadre d'un traitement avec suivi médical
- ☐ Médicament(s) psychotrope(s) hors suivi-médical
- ☐ Substances psychotropes illégales
- ☐ Autres (alcool, café, tabac, etc.)

[]

Quel(s) étai(en)t l'/les objectif(s) de cette prise conjointe et délibérée de substances?

*

Répondre à cette question seulement si les conditions suivantes sont réunies :
La réponse était 'Oui' à la question '9 [Conso5]' (Vous est-il arrivé de prendre conjointement et délibérément plusieurs de ces médicaments ou substances psychotropes?)

Veuillez choisir toutes les réponses qui conviennent :

- ☐ Amplifier un effet (par exemple, prendre plusieurs substances ou médicaments pour le même effet)
- ☐ Contre-balancer un effet (par exemple prendre du café pour contrer les effets d'un somnifère)
- ☐ Autre: ____________

[]

Dans ce qui suit, vous allez pouvoir faire part de vos expériences liées exclusivement à la prise de médicaments psychotropes.

Quels effets ressent(i)ez-vous suite à la prise de ce(s) médicament(s) psychotrope(s)?

*

Répondre à cette question seulement si les conditions suivantes sont réunies :
La réponse était 'Oui' à la question '1 [Conso1]' (Faites-vous ou avez-vous fait l'expérience de prises de médicaments psychotropes?)

Choisissez la réponse appropriée pour chaque élément :

	Tout à fait d'accord	D'accord	Sans avis	Pas d'accord	Pas du tout d'accord
Je me sens plus serein·e, calme	○	○	○	○	○
Je me sens plus relaxé·e en situation sociale	○	○	○	○	○
J'arrive plus facilement à m'endormir	○	○	○	○	○
Je me sens endormi·e	○	○	○	○	○
Je me sens plus compétent·e, performant·e	○	○	○	○	○
Je peux mieux me concentrer	○	○	○	○	○
Je me sens plus en confiance	○	○	○	○	○
Je perds l'appétit	○	○	○	○	○
J'ai des palpitations	○	○	○	○	○
J'ai des nausées	○	○	○	○	○
Je me sens nerveux·euse	○	○	○	○	○
Je me sens énervé·e	○	○	○	○	○
Je me sens agressif·ve	○	○	○	○	○
Je me sens coupable	○	○	○	○	○

[]D'une manière générale, les effets ressentis lors de la prise de médicaments psychotropes correspond(ai)ent à mes attentes
*

Répondre à cette question seulement si les conditions suivantes sont réunies :
La réponse était 'Oui' à la question '1 [Conso1]' (Faites-vous ou avez-vous fait l'expérience de prises de médicaments psychotropes?)

Veuillez sélectionner une seule des propositions suivantes :

- ○ Tout à fait d'accord
- ○ D'accord
- ○ Sans avis
- ○ Pas d'accord
- ○ Pas du tout d'accord

[]Comment vous procur(i)ez-vous ce(s) médicament(s) psychotrope(s)?

*

Répondre à cette question seulement si les conditions suivantes sont réunies :
La réponse était 'Oui' à la question '1 [Conso1]' (Faites-vous ou avez-vous fait l'expérience de prises de médicaments psychotropes?)

Veuillez choisir toutes les réponses qui conviennent :

- ☐ Dans le cadre de mon traitement faisant l'objet d'un suivi médical
- ☐ Donné(s) par un·e membre de ma famille
- ☐ Donné(s) par un·e ami·e
- ☐ Donné(s) par un·e étudiant·e
- ☐ Acheté(s) à une personne de mon entourage
- ☐ Acheté(s) à une personne hors de mon entourage
- ☐ Acheté(s) sur internet
- ☐ Acheté(s) dans une pharmacie / un commerce
- ☐ Autre: ____________

[]Lors de ma toute première prise d'un médicament psychotrope, je l'ai pris... *

Répondre à cette question seulement si les conditions suivantes sont réunies :
La réponse était 'Oui' à la question '1 [Conso1]' (Faites-vous ou avez-vous fait l'expérience de prises de médicaments psychotropes?)

Veuillez choisir toutes les réponses qui conviennent :

- ☐ seul·e
- ☐ avec un·e ami·e·s
- ☐ avec un·e membre de ma famille
- ☐ avec un·e autre étudiant·e
- ☐ avec une personne hors de mon entourage
- ☐ avec un·e professionnel·le de la santé
- ☐ Autre: ____________

[]Pour quelle(s) raison(s), motivation(s) avez-vous pris ce ou ces médicament·s psychotrope·s ? *

Répondre à cette question seulement si les conditions suivantes sont réunies :
La réponse était 'Oui' à la question '1 [Conso1]' (Faites-vous ou avez-vous fait l'expérience de prises de médicaments psychotropes?)

Veuillez choisir toutes les réponses qui conviennent :

- ☐ Sur prescription de mon ou ma médecin dans le cadre d'un traitement
- ☐ Suite à un auto-diagnostic
- ☐ Pour diminuer mon anxiété ou mon stress lié à ma vie quotidienne
- ☐ Pour gérer mon niveau de stress avant un événement académique particulier (examen, épreuve, etc.)
- ☐ Pour améliorer mes performances académiques (concentration, attention, mémoire, etc.)
- ☐ Pour améliorer la qualité de mon sommeil
- ☐ Par curiosité ou pour un usage récréatif
- ☐ Pour faciliter mes relations ou mon intégration sociale
- ☐ Pour gérer mes craintes face à un avenir incertain
- ☐ Autre: ____________

PARTIE C : PERCEPTIONS DE LA PRISE ET PARTAGES D'EXPÉRIENCES

Dans cette partie, vous allez pouvoir faire part de vos expériences liées à la manière dont vous percevez vos pratiques de prises et au regard des autres à votre égard.

[]Informez-vous des personnes sur votre prise de médicaments psychotropes? *

Répondre à cette question seulement si les conditions suivantes sont réunies :
La réponse était 'Oui' à la question '1 [Conso1]' (Faites-vous ou avez-vous fait l’expérience de prises de médicaments psychotropes?)

Veuillez sélectionner une seule des propositions suivantes :

- ○ Oui
- ○ Non

[]A qui parlez-vous de votre prise? *

Répondre à cette question seulement si les conditions suivantes sont réunies :
La réponse était 'Oui' à la question '17 [Rai1]' (Informez-vous des personnes sur votre prise de médicaments psychotropes?)

Veuillez choisir toutes les réponses qui conviennent :

- ☐ J’en parle à ma famille
- ☐ J’en parle à mes ami·e·s
- ☐ J’en parle à d’autres étudiant·e·s
- ☐ J’en parle à des professionnel·le·s de la santé
- ☐ J’en parle à des professionnel·le·s de l'Unil
- ☐ J’en parle à d’autres personnes
- ☐ Je n’en parle à personne

[]Je me sens jugé·e négativement lorsque j’informe des personnes sur ma prise de médicaments psychotropes. *

Répondre à cette question seulement si les conditions suivantes sont réunies :
La réponse était 'Oui' à la question '17 [Rai1]' (Informez-vous des personnes sur votre prise de médicaments psychotropes?)

Veuillez sélectionner une seule des propositions suivantes :

- ○ Tout à fait d'accord
- ○ D'accord
- ○ Sans avis
- ○ Pas d'accord
- ○ Pas du tout d'accord

[]Échangez-vous des informations à propos des médicaments psychotropes avec d’autres étudiant·e·s? *

Répondre à cette question seulement si les conditions suivantes sont réunies :
La réponse était 'Oui' à la question '1 [Conso1]' (Faites-vous ou avez-vous fait l’expérience de prises de médicaments psychotropes?)

Veuillez sélectionner une seule des propositions suivantes :

- Oui
- Non

[]Globalement, le fait d’en parler avec d’autres étudiant·e·s … *

Répondre à cette question seulement si les conditions suivantes sont réunies :
La réponse était 'Oui' à la question '20 [Rai2]' (Échangez-vous des informations à propos des médicaments psychotropes avec d’autres étudiant·e·s?)

Veuillez sélectionner une seule des propositions suivantes :

- …me dissuade généralement d’en consommer
- …m’encourage généralement à en consommer
- …n’a pas d’influence

[]Ma prise de médicaments psychotropes m’inquiète. *

Répondre à cette question seulement si les conditions suivantes sont réunies :
La réponse était 'Oui' à la question '1 [Conso1]' (Faites-vous ou avez-vous fait l’expérience de prises de médicaments psychotropes?)

Veuillez sélectionner une seule des propositions suivantes :

- Tout à fait d'accord
- D'accord
- Sans avis
- Pas d'accord
- Pas du tout d'accord

[]Connaissez-vous des étudiant·e·s qui prennent des médicaments psychotropes ?

*

Veuillez sélectionner une seule des propositions suivantes :

- Oui
- Non

[]De manière générale, quelles sont vos perceptions des étudiant·e·s qui prennent des médicaments psychotropes hors du cadre d'un traitement avec suivi médical? *

Choisissez la réponse appropriée pour chaque élément :

	Tout à fait d'accord	D'accord	Sans avis	Pas d'accord	Pas du tout d'accord
Je pense qu'elles/ils se mettent en danger	○	○	○	○	○
Cela m'inquiète	○	○	○	○	○
Cela me dérange	○	○	○	○	○
Le fait que cela leur fasse du bien le justifie	○	○	○	○	○
Prendre des médicaments psychotropes correspond à un besoin	○	○	○	○	○
Si les médicaments psychotropes augmentent les capacités cognitives, en prendre est injuste par rapport aux autres étudiant·e·s qui n'en prennent pas	○	○	○	○	○

[]Avez-vous déjà songé à essayer un médicament psychotrope ?

*

Répondre à cette question seulement si les conditions suivantes sont réunies :
La réponse était 'Non' à la question '1 [Conso1]' (Faites-vous ou avez-vous fait l'expérience de prises de médicaments psychotropes?)

Veuillez sélectionner une seule des propositions suivantes :

- ○ Oui (dans le cadre d'un suivi médical)
- ○ Oui (hors suivi médical)
- ○ Non

[]Pour quelle(s) raison(s)? *

Répondre à cette question seulement si les conditions suivantes sont réunies :
La réponse était inférieure à 'Non ' à la question '25 [Per3]' (Avez-vous déjà songé à essayer un médicament psychotrope ?)

Veuillez choisir toutes les réponses qui conviennent :

- ☐ Pour diminuer mon anxiété ou mon stress lié à ma vie quotidienne
- ☐ Pour gérer mon niveau de stress avant un événement académique particulier (examen, épreuve, etc.)
- ☐ Pour améliorer mes performances académiques (concentration, attention, mémoire, etc.)
- ☐ Pour améliorer la qualité de mon sommeil
- ☐ Par curiosité ou pour un usage récréatif
- ☐ Pour faciliter mes relations ou mon intégration sociale
- ☐ Par crainte face à un avenir incertain
- ☐ Autre: ____________

[]Pour quelle(s) raison(s)? *

Répondre à cette question seulement si les conditions suivantes sont réunies :
La réponse était 'Non ' à la question '25 [Per3]' (Avez-vous déjà songé à essayer un médicament psychotrope ?)

Veuillez choisir toutes les réponses qui conviennent :

- ☐ Je n’en avais pas envie
- ☐ Par peur des effets secondaires et des conséquences sur la santé
- ☐ Par peur de l’addiction
- ☐ Par difficulté à se procurer la substance (prix, facilité d’obtention)
- ☐ Mes proches m’en dissuadent
- ☐ Mauvaise expérience d’une connaissance
- ☐ Autre: ____________

[]Quelles sont selon vous les raisons qui poussent des étudiant·e·s à consommer des médicaments psychotropes? *

Veuillez choisir toutes les réponses qui conviennent :

- ☐ Sur prescription de leur médecin dans le cadre d’un traitement
- ☐ Suite à un auto-diagnostic
- ☐ Pour diminuer leur anxiété ou leur stress lié à leur vie quotidienne
- ☐ Pour gérer leur niveau de stress avant un événement académique particulier (examen, épreuve, etc.)
- ☐ Pour améliorer leurs performances académiques (concentration, attention, mémoire, etc.)
- ☐ Pour améliorer la qualité de leur sommeil
- ☐ Par curiosité ou pour un usage récréatif
- ☐ Pour faciliter leurs relations ou leur intégration sociale
- ☐ Par craintes face à un avenir incertain
- ☐ Autre: ____________

[]Est-ce qu’une/des personne(s) vous a/ont déjà proposé d’essayer un médicament psychotrope? *

Veuillez sélectionner une seule des propositions suivantes :

- ○ Oui
- ○ Non

[]De qui s'agit-il? *

Répondre à cette question seulement si les conditions suivantes sont réunies :
La réponse était 'Oui' à la question '29 [Per5]' (Est-ce qu’une/des personne(s) vous a/ont déjà proposé d’essayer un médicament psychotrope?)

Veuillez choisir toutes les réponses qui conviennent :

- [ ] mon/ma médecin
- [ ] un/des membre(s) de ma famille
- [ ] un·e/des ami·e·s
- [ ] un·e/des étudiant·e·s
- [ ] une/des personne(s) de mon entourage
- [ ] Autre: ______

[]Avez-vous accepté? *

Répondre à cette question seulement si les conditions suivantes sont réunies :
La réponse était 'Oui' à la question '29 [Per5]' (Est-ce qu’une/des personne(s) vous a/ont déjà proposé d’essayer un médicament psychotrope?)

Veuillez sélectionner une seule des propositions suivantes :

- ○ Oui
- ○ Non

PARTIE D : QUALITE DE VIE & BIEN ETRE

Vous pouvez maintenant vous exprimer sur la façon dont vous percevez actuellement votre qualité de vie et votre bien être.

[]

Actuellement, en ce qui concerne ma qualité de vie et mon bien-être...

*****

Choisissez la réponse appropriée pour chaque élément :

	Tout à fait d'accord	D'accord	Sans avis	Pas d'accord	Pas du tout d'accord
Je pense que ma vie correspond à mes idéaux dans la plupart des domaines	○	○	○	○	○
Je suis globalement satisfait·e de ma vie	○	○	○	○	○
Je pense que je suis une personne de valeurs	○	○	○	○	○
Je suis capable de faire les choses aussi bien que la majorité des gens	○	○	○	○	○
J'ai peu de raisons d'être fier·ère de moi	○	○	○	○	○
Je me sens épanoui·e dans mes études	○	○	○	○	○
Je me sens soutenu·e par les membres de mon entourage	○	○	○	○	○
Je suis satisfait·e de mes relations sociales	○	○	○	○	○
Je me sens capable de résoudre par moi-même les problèmes que je rencontre au quotidien	○	○	○	○	○
Je peux gérer ma vie comme je l'entends	○	○	○	○	○

[]De manière générale, je considère mon état de santé comme... *

Veuillez sélectionner une seule des propositions suivantes :

○ Très bon

○ Bon

○ Mauvais

○ Très mauvais

[]Quel(s) type(s) de difficultés rencontrez-vous dans vos études? *

Choisissez la réponse appropriée pour chaque élément :

	Tout à fait d'accord	D'accord	Sans avis	Pas d'accord	Pas du tout d'accord
La matière enseignée dans les cours est trop difficile, j’ai trop de travail, je suis dépassé·e par le niveau exigé.	○	○	○	○	○
Mon horaire est trop chargé ou mal réparti, certains cours se chevauchent.	○	○	○	○	○
J’ai des difficultés à adapter mes méthodes de travail (gestion du temps, prise de notes, autodiscipline, autonomie, etc.).	○	○	○	○	○
J’ai vécu des échecs scolaires et cela engendre chez moi du stress ou de l’anxiété.	○	○	○	○	○
Je ne suis plus intéressé·e par mes études, je suis démotivé·e par certains cours.	○	○	○	○	○
Je me sens isolé.e socialement, j’ai de la peine à me faire des ami·e·s, la compétition est trop intense dans ma faculté.	○	○	○	○	○
Je rencontre des difficultés d'intégration liées à mon appartenance religieuse, culturelle, linguistique ou sexuelle.	○	○	○	○	○
J'ai des problèmes dans ma vie privée qui génèrent de l'inquiétude, du stress ou de l'anxiété.	○	○	○	○	○

[]Avez-vous un ou plusieurs handicap(s)? *

Veuillez sélectionner une seule des propositions suivantes :

○ Oui

○ Non

[]De quel(s) handicap(s) s’agit-il? *

Répondre à cette question seulement si les conditions suivantes sont réunies :
La réponse était 'Oui' à la question '35 [Qua3]' (Avez-vous un ou plusieurs handicap(s)?)

Veuillez choisir toutes les réponses qui conviennent :

- ☐ Mobilité réduite
- ☐ Diminution de la capacité auditive
- ☐ Dyslexie, dysorthographie
- ☐ TDAH (trouble de l’attention avec ou sans hyperactivité)
- ☐ Autre: ______________

[]Ce(s) handicap(s) constitue(nt)-t-il(s) une difficulté dans vos études? *

Répondre à cette question seulement si les conditions suivantes sont réunies :
La réponse était 'Oui' à la question '35 [Qua3]' (Avez-vous un ou plusieurs handicap(s)?)

Veuillez sélectionner une seule des propositions suivantes :

- ○ oui, au quotidien
- ○ oui, parfois
- ○ non, jamais

[]Considérez-vous avoir des comportements dits « à risques »? *

Choisissez la réponse appropriée pour chaque élément :

	oui	plutôt oui	plutôt non	non
Pratiques sexuelles à risques	○	○	○	○
Entretien de relations sociales pesantes émotionnellement	○	○	○	○
Mise en danger dans une activité sportive (prise de produits dopants, manque de matériel et/ou de mesures de sécurité, etc.)	○	○	○	○
Alimentation problématique (sur/sous/mauvaise alimentation)	○	○	○	○
Trop de temps sur mon smartphone, hyper connectivité (internet, réseaux sociaux, jeux vidéos, etc.)	○	○	○	○

[]

Quels dispositifs d'aides, dont vous pouvez bénéficier à l'UNIL, connaissez-vous?

*****

Veuillez choisir toutes les réponses qui conviennent :

- ☐ Service des Affaires Sociales et de la Mobilité étudiante (SASM)
- ☐ Service d'Orientation et de Conseil (SOC)
- ☐ Accueil Santé
- ☐ Consultation psycho-thérapeutique
- ☐ Ateliers (gestion du stress, préparation des examens, mieux gérer le temps, etc.)
- ☐ Associations d'étudiant·e·s
- ☐ Aucun

[]A quel(s) dispositif(s) d'aide avez-vous déjà eu recours? *

Veuillez choisir toutes les réponses qui conviennent :

- ☐ Service des Affaires Sociales et de la Mobilité étudiante (SASM)
- ☐ Service d'Orientation et de Conseil (SOC)
- ☐ Accueil Santé
- ☐ Consultation psycho-thérapeutique
- ☐ Ateliers (gestion du stress, préparation des examens, mieux gérer le temps, etc.)
- ☐ Associations d'étudiant·e·s
- ☐ Aucun
- ☐ Autre: ____________________

PROFILS

Nous allons maintenant vous poser des questions concernant votre profil.

[]Quelle est votre configuration familiale ? Je vis... *

Veuillez choisir toutes les réponses qui conviennent :

- [ ] seul·e
- [ ] en collocation
- [ ] chez mon/mes parent(s)
- [ ] chez un·e membre de ma famille
- [ ] avec mon/mes enfant(s)
- [ ] en couple
- [ ] dans une famille d'accueil
- [ ] dans une institution sociale (foyer social, établissement médico-social)
- [ ] sans domicile
- [ ] Autre: ____________

[]Comment définiriez-vous votre situation financière actuelle ? *

Veuillez sélectionner une seule des propositions suivantes :

- ○ Très bonne
- ○ Bonne
- ○ Mauvaise
- ○ Très mauvaise

[]Êtes-vous au bénéfice d'une bourse d'étude? *

Veuillez sélectionner une seule des propositions suivantes :

- ○ Oui
- ○ Non

[]Travaillez-vous en dehors de vos études de manière rémunérée ? *

Veuillez sélectionner une seule des propositions suivantes :

- ○ Oui
- ○ Non

[]Combien d'heures par semaine consacrez-vous en moyenne sur un semestre à ce travail rémunéré? *

Répondre à cette question seulement si les conditions suivantes sont réunies :
La réponse était 'Oui' à la question '44 [Pro4]' (Travaillez-vous en dehors de vos études de manière rémunérée ?)

Veuillez sélectionner une seule des propositions suivantes :

- ◯ 1 à 4 heures par semaine
- ◯ 5 à 10 heures par semaines
- ◯ 11 à 20 heures par semaine
- ◯ 21 à 40 heures par semaine
- ◯ Plus de 40 heures par semaine

[]Quel est votre sexe ? *

Veuillez sélectionner une seule des propositions suivantes :

- ◯ Femme
- ◯ Homme
- ◯ Autre

[]Quel est votre âge ? *

Veuillez sélectionner une seule des propositions suivantes :

- ◯ ≤ 18 ans
- ◯ 19 – 24
- ◯ 25 – 30
- ◯ 31 - 40
- ◯ 41 et plus

[]Quelle est votre filière d’étude ? *

Veuillez sélectionner une seule des propositions suivantes :

- ◯ Faculté des Sciences Sociales et Politiques
- ◯ Faculté de Biologie et de Médecine
- ◯ Faculté des Géosciences et de l’Environnement
- ◯ Faculté des Lettres
- ◯ Faculté de Droit, des Sciences Criminelles et d’Administration publique
- ◯ Faculté des Hautes Etudes Commerciales
- ◯ Faculté de Théologie et de Sciences des Religions

[]A quel niveau de votre cursus d'étude êtes-vous ? *

Veuillez sélectionner une seule des propositions suivantes :

- ○ 1ère année de Bachelor
- ○ 2e et 3e années de Bachelor
- ○ Master

Vous êtes arrivé·e à la fin de ce questionnaire. Nous vous sommes infiniment reconnaissant·e·s d'avoir pris le temps d'y répondre.

Encore une ultime remarque : nous recherchons des participant·e·s qui souhaiteraient dans une deuxième étape s'entretenir avec nous pour approfondir certains thèmes. Si vous êtes intéressé·e à participer, vous pouvez nous le faire savoir en envoyant un message à l'adresse ci-jointe melody.pralong@unil.ch et indiquer : *J'accepte d'être contacté·e pour un entretien à des fins de recherche sur le thème de la prise de médicaments et de substances psychotropes par les étudiant.e.s.*

Mille mercis !

10/04/2020 – 15:07

Envoyer votre questionnaire.
Merci d'avoir complété ce questionnaire.
